# Spontaneous coronary artery dissection (SCAD): A contemporary review

**DOI:** 10.1002/clc.24236

**Published:** 2024-06-11

**Authors:** Sophie Offen, Cathevine Yang, Jacqueline Saw

**Affiliations:** ^1^ Division of Cardiology Vancouver General Hospital, University of British Columbia Vancouver British Columbia Canada

**Keywords:** FMD—fibromuscular dysplasia, IVUS—intravascular ultrasound, NSTEMI—non‐ST elevation myocardial infarction, OCT—optical coherence tomography, SCAD—spontaneous coronary artery dissection, STEMI—ST‐elevation myocardial infarction

## Abstract

Spontaneous coronary artery dissection (SCAD) is an increasingly recognized cause of myocardial infarction that most frequently affects younger women, making it an important cause of morbidity and mortality within these demographics. The evolution of intracoronary imaging, improved diagnosis with coronary angiography, and ongoing research efforts and attention via social media, has led to increasing recognition of this previously underdiagnosed condition. In this review, we provide a summary of the current body of knowledge, as well as focused updates on the pathogenesis of SCAD, insights on genetic susceptibility, contemporary diagnostic tools, and immediate, short‐ and long‐term management.

## Graphical abstract

Spontaneous Coronary Artery Dissection (SCAD) is an increasingly recognized cause of myocardial infarction that most frequently affects younger women, making it an important cause of morbidity and mortality within these demographics. The evolution of intracoronary imaging (C ‐ OCT image of IMH), improved diagnosis with coronary angiography (B ‐ Type 2 A SCAD in an obtuse marginal artery), and ongoing research efforts and attention via social media, has led to increasing recognition of this previously underdiagnosed condition (A ‐ IMH as the important pathophysiologic mechanism in SCAD).

OCT, optical coherence tomography; IMH, intramural hematoma; SCAD, Spontaneous Coronary Artery Dissection.

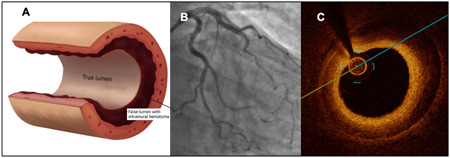

## 1 INTRODUCTION

Spontaneous coronary artery dissection (SCAD) is an infrequent, yet important cause of acute coronary syndrome (ACS) and sudden cardiac death (SCD). It is defined as a non‐atherosclerotic, non‐traumatic and non‐iatrogenic separation of the coronary arterial wall by intramural haematoma, resulting in a false lumen with or without intimal tear. Compression of the arterial lumen by intramural haematoma can lead to compromise of antegrade coronary perfusion and result in myocardial ischemia or infarction.^1^ SCAD is more common both in women and in younger patients, making it an important cause of morbidity and mortality within these demographics. Once believed to be a rare phenomenon, a combination of the evolution of intracoronary imaging, improved diagnosis on coronary angiography, coupled with ongoing research efforts and attention via social media, has led to increasing recognition of this condition. In fact, among women less than 50 years old, SCAD may account for nearly a quarter of all cases of acute myocardial infarction (AMI).^2^

Yet despite raised awareness and recent insights into the epidemiology, clinical and angiographic characteristics of SCAD, high‐quality evidence‐based guidance for its management remains lacking and currently available consensus guidelines still rely heavily on expert opinion. The Canadian SCAD study is an ongoing large multicenter, prospective, observational study in North America, that continues to enroll patients to improve our understanding of the natural history of this condition. The 3‐year outcomes of this study were published in 2022.^3^ Initiatives such as this have informed and justified concurrent randomized control trials evaluating the efficacy of pharmacotherapy in SCAD. Both will contribute to an evolved understanding and best practices required to improve outcomes in this not‐so‐uncommon condition.

## 2 EPIDEMIOLOGY

The index of suspicion for SCAD should be heightened in young women with few traditional cardiovascular risk factors presenting with ACS. In the Canadian SCAD (CanSCAD) cohort study, mean age at presentation was 51.8 ± 10.2, and 88.5% were women (55% post‐menopausal).^4^ However, SCAD can affect patients over a wide age range, from 24 to 89 years in the CanSCAD cohort. Approximately one‐third of these patients had hypertension, and a third had no cardiac risk factors. Other relevant clinical history can include migraine (32.5%), anxiety (19.7%), and depression (19.5%). Potential predisposing conditions occurred in 49.9% of which fibromuscular dysplasia (FMD) was the most common; multifocal FMD was diagnosed in 31.1% of the overall cohort, and 56.7% of those who had complete FMD screening. Precipitating stressors (emotional and physical) at the time of SCAD presentation were frequently recalled (66.4%), with a significant emotional stress being most common (50.3%).^4^

Although significantly more frequent amongst young women, men can also present with SCAD. In a recent analysis of 1173 patients in the CanSCAD study, 10% were men and were notably younger at presentation than women with SCAD (mean age 49.4 ± 9.6 years vs. 52.0 ± 10.6 years; *p* = .01). Men were also less likely to have FMD (27.8% vs. 52.7%; *p* = .001), depression (9.8% vs. 20.2%; *p* = .005) or emotional stress (35.0% vs. 59.3%; *p* < .001). However, they were more likely to report isometric physical stress (40.2% vs. 24.0%; *p* = .007) as a precipitant for their acute SCAD event.^5^

## 3 PATHOPHYSIOLOGY

**Figure 1:**
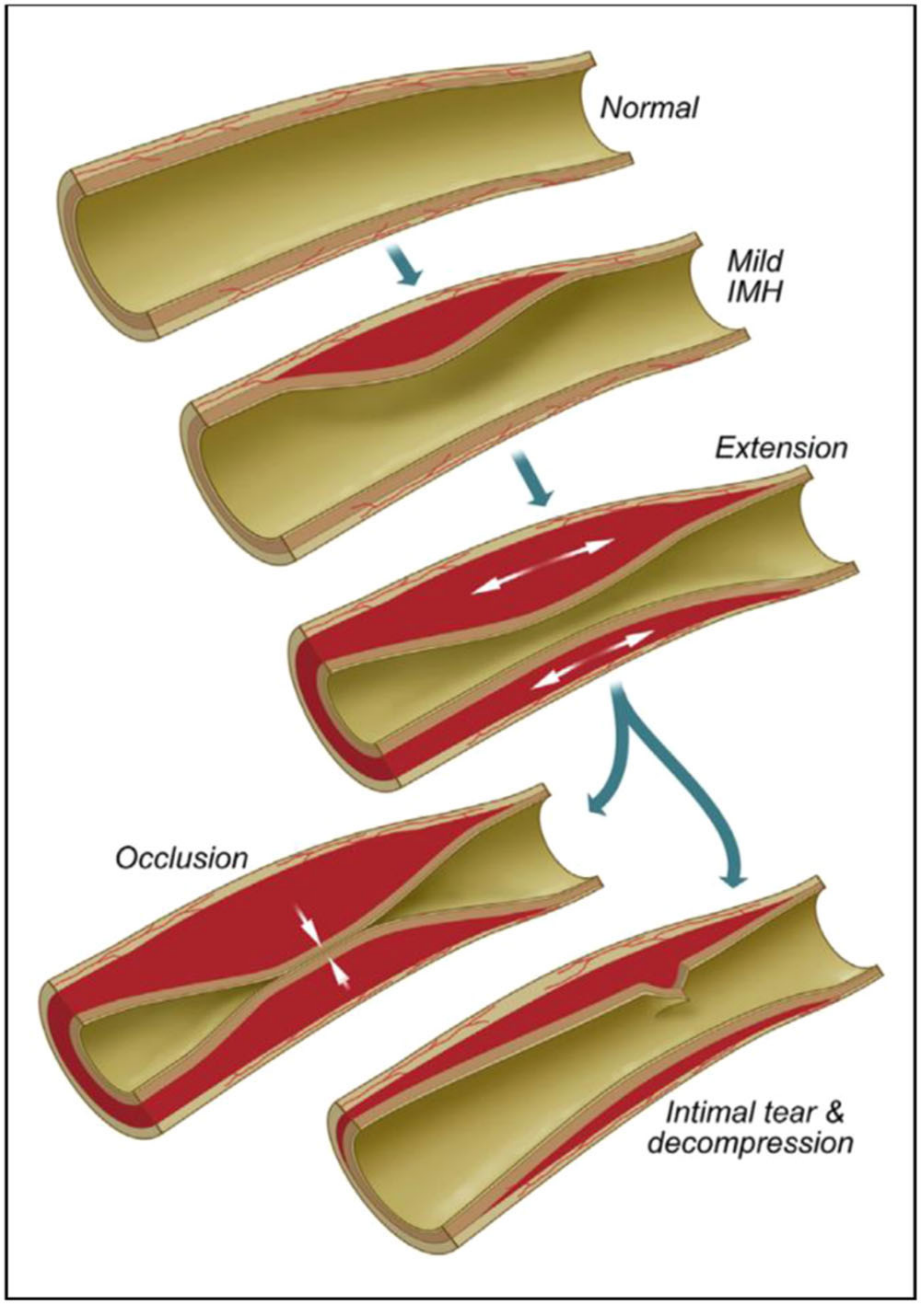
Hypothesized formation of intramural hematoma according to the “outside‐in” hypothesis. Reproduced from Hayes et al. JACC 2020.^6^

Whilst the underlying pathophysiology of SCAD remains incompletely understood, several hypotheses have been previously suggested to describe the development of intramural hematoma (IMH) and associated separation of the intima or intima‐mediated complex from the underlying vessel leading to compression of the true lumen.^6^ The “outside‐in,” as opposed to the “inside‐out,” hypothesis has gained traction based upon autopsy and intravascular imaging studies which have demonstrated that in most SCAD cases IMH arises de novo in the media, possibly due to disruption of the vasa vasorum (Figure 1). Insights from OCT imaging suggest that the false lumen is pressurized before the development of fenestration, which subsequently arise from rupture of the false into the true lumen.^7^ Longitudinal studies also support this hypothesis with serial angiography demonstrating IMH precedes intimal dissection,^8^ and studies of coronary histopathology which found no evidence of endothelial or intimal injury.^9^

## 4 DISEASE ASSOCIATIONS AND GENETICS

In over 50 percent of SCAD cases, there are identifiable cardiocirculatory stressors that may increase the risk of acute SCAD events; this is especially true if there is a pre‐existing arteriopathy, such as FMD.^10^ FMD is the most commonly observed predisposing arteriopathy in SCAD patients and extra‐coronary FMD can be diagnosed on catheter‐based angiography of renal and iliac arteries during coronary angiographies or with computed tomography angiography.^11^, ^12^ Since we reported the first case series of concomitant SCAD and extra‐coronary FMD in 2011^13^ a high prevalence (72%–86%) of FMD has been observed in routinely screened SCAD cohorts.^10^ The prevalence of extra‐coronary FMD in 346 patients from the CanSCAD cohort who had completed screening for renal, iliac and cerebral FMD was 72.3%.^14^ Other causes of SCAD are much less frequent, but can include connective tissue disorders and systemic inflammatory diseases (Table 1).

**Table 1:** Potential predisposing and precipitating factors for SCAD.

Predisposing causes
Fibromuscular dysplasia
Pregnancy‐related: antepartum, early post‐partum, late post‐partum, very late post‐partum
Recurrent pregnancies: multiparity or multigravida
Connective tissue disorder: Marfan syndrome, Loeys‐ Dietz syndrome, Ehler‐Danlos syndrome type 4, cystic medial necrosis, alpha‐1 antitrypsin deficiency, polycystic kidney disease
Systemic inflammatory disease: systemic lupus erythematosus, Crohn's disease, ulcerative colitis, polyarteritis nodosa, sarcoidosis, Churg‐Strauss syndrome, Wegener's granulomatosis, rheumatoid arthritis, Kawasaki, giant cell arteritis, celiac disease
Hormonal therapy: oral contraceptive, estrogen, progesterone, beta‐HCG, testosterone, corticosteroids
Coronary artery spasm
Idiopathic
**Precipitating stressors**
Intense exercises (isometric or aerobic activities)
Intense emotional stress
Labor and delivery
Intense Valsalva‐type activities (e.g., retching, vomiting, bowel movement, coughing)
Recreational drugs (e.g., cocaine, amphetamines, methamphetamines)
Intense hormonal therapy (e.g., beta‐HCG injections, corticosteroids injections)

*Note*: Adapted from Saw et al. JACC 2016.^1^

Abbreviations: HCG, human chorionic gonadotropin; SCAD, spontaneous coronary artery dissection.

Pregnancy‐associated SCAD (P‐SCAD) is another important cause of AMI in younger women, accounting for up to 43% of all pregnancy‐associated AMI.^6^ In these patients, dissection may be a consequence of increased physiological hemodynamic stresses or from hormonal effects weakening the coronary arterial wall.^15^ The majority occur post‐partum and may be associated with more severe disease presentations a higher risk for recurrence. SCAD has also been associated with other hormonal changes including in vitro fertilization, oral contraception and hormone replacement therapy. However, the mechanistic effects of sex hormones on vascular smooth muscle which may increase the risk of SCAD remains to be elucidated.

There is also increasing recognition of the genetic susceptibility to SCAD. Genome‐wide association studies have previously have identified common single nucleotide variants associated with disease susceptibility.^16^, ^17^ A recent meta‐analysis looking at 16 risk loci for SCAD, most involved in regulating vascular smooth muscle cells and artery fibroblasts, demonstrated high polygenic heritability for SCAD as well as a genome‐wide negative correlation with atherosclerotic coronary artery disease.^18^

## 5 CLINICAL PRESENTATION AND DIAGNOSIS

The clinical presentation of patients with SCAD is similar to that of atherosclerotic ACS, with the majority of patients presenting with chest pain, ECG abnormalities and biomarker elevation to denote an ACS. A high index of suspicion should, therefore, be raised in patients, particularly young women, with a paucity of traditional cardiovascular risk factors.^19^, ^20^ In the CanSCAD study, 69.9% had non‐ST‐segment elevation MI (NSTEMI), 29.7% had ST‐segment elevation MI (STEMI) and the most common presenting symptom was chest discomfort (91.5%). Other presenting symptoms can include nausea or vomiting, diaphoresis, dyspnea or lightheadedness.^21^ A small proportion (8.1%) present with ventricular tachycardia or fibrillation.^4^ And even less frequently, SCAD can present with cardiogenic shock or SCD (<1% based on pathology series).^22^, ^23^ Elevation of serum biomarkers and ischemic ECG changes are also common, as are wall motion abnormalities (>80% cases).^4^ The latter can be very helpful to aid in the angiographic recognition of SCAD.

**Figure 2:**
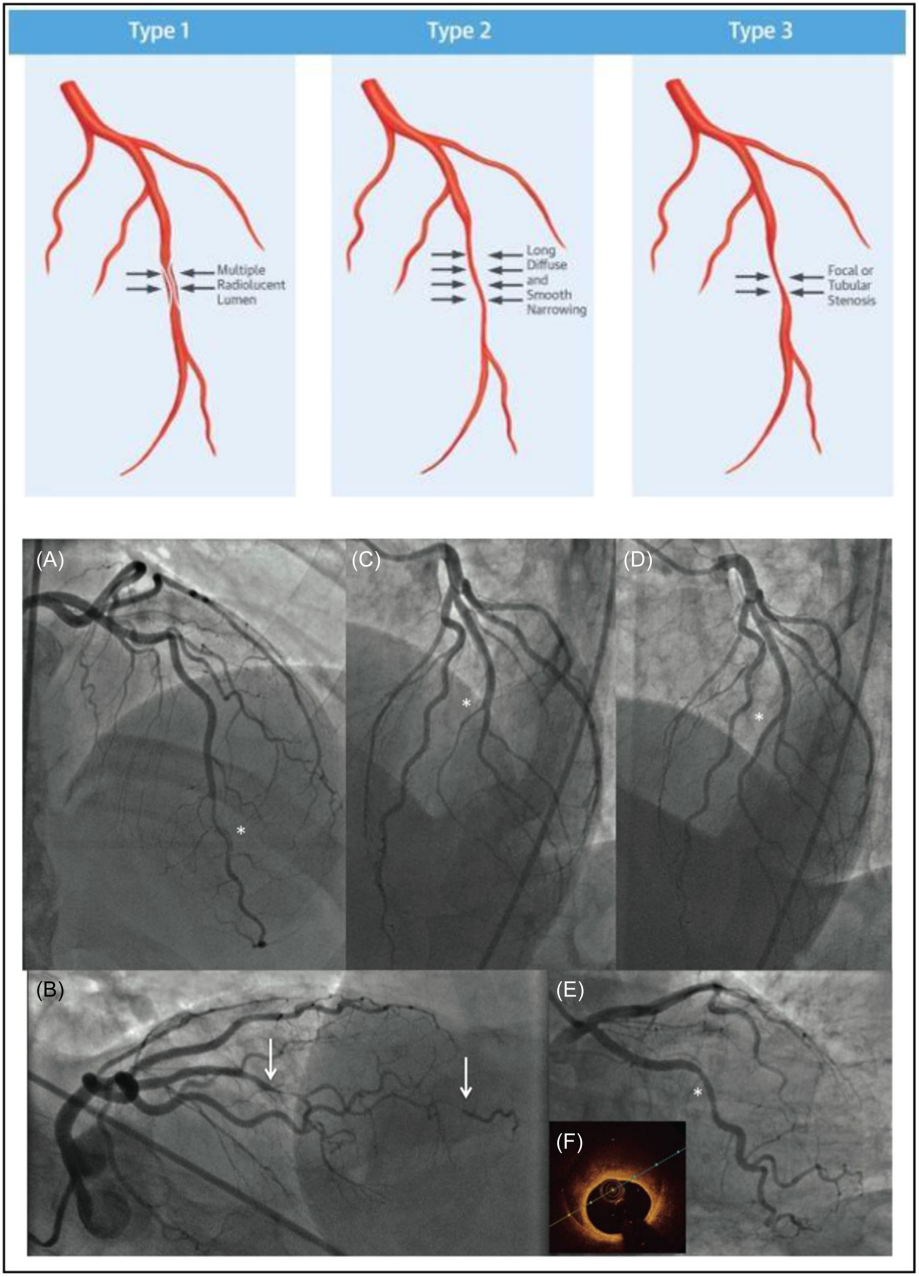
Angiographic classification of SCAD; (A) Type 1 spontaneous coronary artery dissection (SCAD) of distal left anterior descending (LAD) artery with staining of artery wall (asterisk). (B) Type 2A SCAD of mid‐distal LAD (between arrows). (C) Type 2B SCAD of diagonal branch (asterisk), which healed 1 year later (asterisk in D). (E) Type 3 SCAD of mid‐ circumflex artery (asterisk), with corresponding optical coherence tomography showing intramural hematoma in (F). Adapted from Saw et al. JACC 2016.^1^

The diagnosis of SCAD is routinely made by invasive coronary angiography, with early and accurate diagnosis of SCAD enabling management and further investigation pathways which differ from atherosclerotic disease. The angiographic classification of SCAD describes three lesion subtypes (Figure 2). Type 1 is the pathognomonic appearance of arterial wall contrast staining with multiple radiolucent lumens. Type 2 is a diffuse stenosis of varying severity and length which may be bordered by normal artery segments proximal and distal to the IMH (Type 2A variant) or extending to the apical tip of the artery (Type 2B). Type 3 describes a focal or tubular stenosis (typically <20 mm) that mimics atherosclerosis and requires intracoronary imaging to confirm diagnosis.^12^ The most common angiographic appearance is Type 2, and 60.2% of patients in the CanSCAD study had this subtype; 34.2% Type 2A and 25.9% Type 2B. Type 1 was present in 29% and Type 3 was least common, in only 10.8%. With regard to coronary distribution, the left anterior descending artery (LAD) was the most frequently involved (52%) and the left main the least (1.5%). Involvement of the proximal segments occurred in only 7.6% and in the majority, SCAD affected only a single coronary segment (74.8%).^4^

More recently, the use of intracoronary imaging (either optical coherence tomography [OCT] or intravascular ultrasound [IVUS]) has been used as an additional tool to improve SCAD diagnosis, with both modalities able to image the layers of the arterial wall. Whilst OCT has superior spatial resolution (10–20 µm) compared to IVUS (150 µm), IVUS has comparatively better penetration. OCT is, therefore, the superior modality for visualizing intimal tears, intraluminal thrombi and IMH, despite being limited by depth of penetration. IVUS provides deeper vessel visualization allowing improved appreciation of the extent of IMH and notably does not require contrast administration. Whilst both OCT and IVUS provide complementary details to aid in the diagnosis of SCAD, a combination of availability, cost and operator skill limit their more widespread use, as does the risk of potential complications. In a recently reported analysis of the 70 of 1306 patients in the CanSCAD study who had OCT or IVUS performed at acute presentation, 8.6% had complications related to intracoronary imaging. Due to the potential risk of contrast injection potentiating dissection with the use of OCT, high definition IVUS may be preferred for use in occlusive lesions where the SCAD diagnosis remains uncertain despite conventional angiography, or in cases when percutaneous coronary intervention (PCI) is required.

## 6 MANAGEMENT CONSIDERATIONS

### 6.1 Conservative management

Whilst there have been no prospective studies to‐date evaluating the optimal revascularization strategy in SCAD, observational data have indicated that a conservative approach is preferable. This recommendation relies on observations that the majority of SCAD lesions heal spontaneously in the weeks to months after conservative management of the index event, and that revascularization is associated with high failure rates,^24^, ^25^, ^26^ as well as major adverse cardiovascular event (MACE) both in‐hospital and during follow‐up.^27^ Furthermore, a recent meta‐analysis of studies comparing the efficacy and safety of a conservative approach demonstrated similar outcomes and lower likelihood of the need for target vessel revascularization when compared to invasive revascularization.^28^

**Figure 3:**
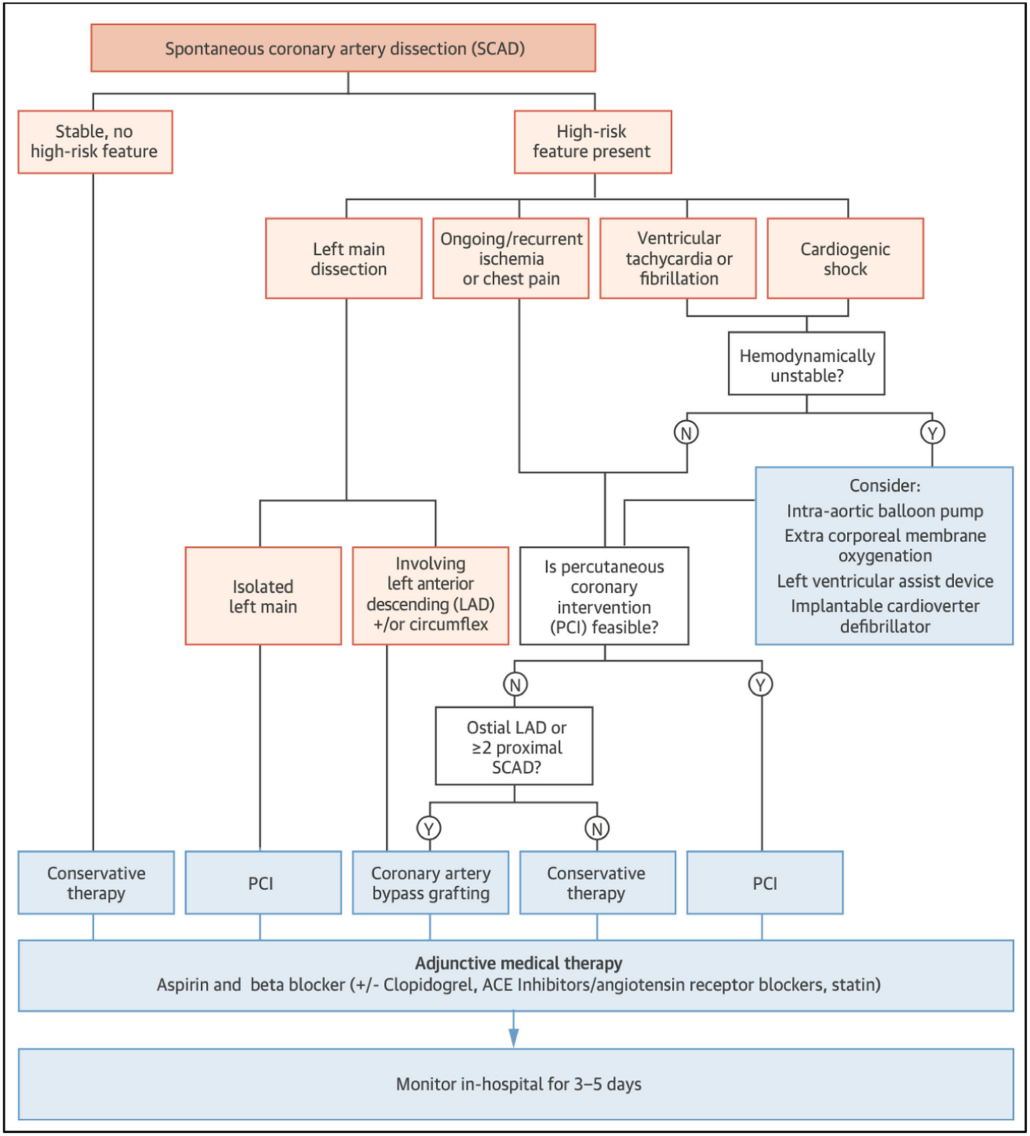
Suggested management algorithm for SCAD. Reproduced with permission from Saw et al. JACC 2016.^1^

The currently available consensus statements, therefore, advocate a conservative strategy for the management of acute SCAD in the majority of patients.^29^ Notable exceptions are patients with ongoing ischemia, haemodynamic or electrical instability or left main dissection who may require urgent revascularization with PCI or coronary artery bypass grafting (CABG). A proposed management algorithm for the acute treatment of SCAD is depicted in Figure 3. In the CanSCAD study the majority of patients were managed conservatively (86.4%), with only 2.3% requiring subsequent revascularization (2% with PCI and 0.3% with CABG).^3^, ^4^ The risk of early complications, including recurrent MI, in conservatively managed patients has led experts to suggest an extended period of monitoring in‐hospital for conservatively managed SCAD patients.^1^, ^6^, ^29^ Typically this is 3–5 days, depending on symptoms and the location of SCAD.^29^

### 6.2 Considerations for invasive revascularization

Preference for a conservative strategy is also influenced by case series reporting on the poor technical success of PCI in SCAD. In the CanSCAD study technical success rates with PCI were relatively poor; in 30.1% PCI was unsuccessful, 40.8% partially successful and in 32% there was propagation of SCAD during PCI.^3^, ^4^ Given the underlying frailty and disrupted integrity of arterial wall, PCI can result in iatrogenic dissection and/or extension of dissection. It is, therefore, the opinion of the authors that to enhance the technical success of PCI and avoid potential complications, a number of strategies should be employed by the interventional cardiologist as depicted in Table 2. Preference for the femoral approach, both for diagnostic angiography and PCI in SCAD, is based upon a potentially threefold higher rate of iatrogenic dissection with a radial approach.^24^ With either approach, meticulous technique and care with catheter engagement is paramount, and use of hand injections as opposed to a power injector is also preferred to best control the hydraulic force of dye injected. The use of longer stents can provide adequate coverage of both proximal and distal edges of the dissected arterial segment. Alternatively, for longer lesions which require multiple stents, a multi‐step approach of first stenting the distal edge, followed by the proximal edge, and finally the middle segment, may be useful in preventing propagation of IMH.^30^

**Table 2:** Challenges and suggestions for PCI in SCAD.

Challenges during PCI of SCAD
Risk of iatrogenic catheter‐induced dissection
Difficulty advancing coronary wire into distal true lumen
Propagating IMH anterograde and retrograde with angioplasty/stenting, extending dissection and further compromising true lumen arterial flow
Propagating IMH anterograde and retrograde with angioplasty/stenting, extending dissection and further compromising true lumen arterial flow
Dissection tends to extend into distal arteries, which are too small for stents
Often extensive dissected segments require long stents, increasing stent restenosis
Risk of stent malapposition after resorption of IMH, with risk of late stent thrombosis
Challenges during PCI of SCAD
Risk of iatrogenic catheter‐induced dissection
Difficulty advancing coronary wire into distal true lumen
**Suggestions if PCI is pursued for SCAD**
Meticulous guide catheter manipulation, preferably through femoral access approach
OCT/IVUS guidance to ensure wire in true lumen (or over‐the‐wire catheter injections) and optimize stent apposition
Long stents covering 5–10 mm of proximal and distal edges of IMH
Placing short stents at proximal and distal edges first, before placing long stent in the middle
Consider bioabsorbable stents (temporary scaffold to avoid long‐ term malapposition)
Possible and careful use of cutting balloon (to fenestrate IMH)
Consider follow‐up OCT to assess for malapposed/uncovered struts before stopping DAPT

*Note*: Adapted from Saw et al. JACC 2016.^1^

Abbreviations: DAPT, dual antiplatelet therapy; IMH, intramural hematoma; IVUS, intravascular ultrasound; PCI, percutaneous coronary intervention.

The available literature on the role of CABG in SCAD is limited to case series and retrospective observational data but surgical revascularization may act as a bridge to recovery in SCAD patients not amenable to conservative or PCI options. These patients have mostly shown good acute survival,^25^ but suboptimal longer‐term outcomes with poor graft patency (only 27% in one small series)^31^ likely due to healing of dissected SCAD segments. Therefore, CABG should only be considered a potential revascularization strategy for patients with left main dissection, extensive dissection involving the proximal coronary arteries or in patients in whom PCI is not feasible when ischemia is refractory. Similarly, evidence for the use of therapies supportive of left ventricular function or persistent ischemia in SCAD, such as intraaortic balloon pumps, Impella devices or extracorporeal membrane oxygenation, are limited to case reports. These therapies may be considered in cases of SCAD with cardiogenic shock as a bridge to recovery, CABG or transplantation and in line with current society guidelines in this area.^31^

### 6.3 Pharmacologic therapy

Like the invasive management of SCAD, there remains no evidence‐based guidelines to guide the medical treatment of SCAD patients either in the acute, or in the long‐term setting. It is unknown whether the standard guidelines‐directed medical therapy for patients with atherosclerotic disease and ACS apply to individuals with SCAD, as the latter has never been specifically studied, and based on the pathophysiology there are likely to be core differences in their management. Thus, current strategies for medical therapy in SCAD are largely based on expert opinions from observational series.

The key medications administered in SCAD patients are beta‐blockers and antiplatelet therapy. Theoretically at least, the administration of beta‐blockade in SCAD patients is generally thought to reduce coronary arterial wall sheer stress, similar to their therapeutic action in patients with aortic dissection. Strengthening this theoretical benefit of beta‐blocker was the demonstration of a lower risk for the recurrence of SCAD (hazard ratio: 0.36, *p* = .004) in a Vancouver series of 327 patients.^32^

Similarly, the distinct pathophysiologic mechanisms of SCAD from those of atherosclerotic ACS suggest the rationale and potential risks of using standard ACS therapies in SCAD patients. For those undergoing PCI, a standard dual antiplatelet regimen (DAPT) with aspirin and P2Y12‐inhibitor should be administered.^33^ The majority of SCAD patients, however, are managed conservatively and there is controversy regarding the benefit of DAPT versus aspirin alone in this cohort. The recent DISCO study retrospectively compared DAPT to single antiplatelet therapy in 199 conservatively managed SCAD patients and reported significantly higher rates of MACEs in those discharged on DAPT.^34^ However, 14 of the 15 in‐hospital MACE events were due to unplanned revascularization with PCI leading to questionable categorization of these patients as “conservatively managed.”^35^ In the significantly larger, prospectively enrolled, Canadian SCAD cohort study of 750 patients, 67.4% were on DAPT at the time of discharge and 28.2% at 1 year. When patients who were managed with PCI at any point during their hospital admission were excluded, the use of P2Y12 inhibitors was not a predictor of worse in‐hospital or longer‐term outcomes. Similar to currently published expert consensus documents, we prescribe clopidogrel as the P2Y12 inhibitor of choice (preferred over ticagrelor or prasugrel due to the lower bleeding risk), limited to a duration of 1–3 months in conservatively managed SCAD patients.^1^ Most experts recommend aspirin for at least 1 year, and typically indefinitely following a SCAD event, in the absence of any contraindications.^29^

Renin–angiotensin system antagonists (ACE‐inhibitors) are usually administered in cases of LV dysfunction or hypertension and statins are reserved for patients with preexisting dyslipidemia or concomitant atherosclerotic disease, both according to relevant society guidelines.

### 6.4 Post‐discharge recommendations

As lifestyle and psychological stressors could affect SCAD recurrence, some combination of cardiac rehabilitation, psychosocial counseling, and peer support are likely beneficial in SCAD patients. Therefore, all patients should be referred to dedicated and individualized cardiac rehabilitation programs to facilitate safe return to physical activities within the recommended limitations following a SCAD event. These dedicated programs are both safe and associated with lower long‐term cardiovascular events.^36^, ^37^ Patients are advised to avoid heavy weightlifting (empiric threshold for women is ~30 lbs and men is ~50 lbs) and to have lower target heart rate (50%–70% heart rate reserve) and systolic blood pressure of <130 mmHg with exercise during rehabilitation after SCAD event.

### 6.5 Prognosis

Long‐term survival in SCAD is excellent, with low rates of mortality (0.8%) out to 3 years in the CanSCAD study cohort, the majority of whom were treated conservatively.^3^ Rates of recurrent MI and de novo recurrent SCAD were similarly low (9.9% and 2.4%, respectively), compared to previously published smaller, retrospective cohorts. These findings may have been due also to the high rate of aspirin (93.7%) and beta‐blocker (84.3%) administration in the CanSCAD study. Notably, pregnancy‐associated SCAD was associated with increased in‐hospital, 30‐day and 3‐year event rates, consistent with other reports and denoting that this entity is likely a more severe phenotype. Other independent predictors of 3‐year MACE were genetic disorders and extra‐coronary FMD.

Despite a relatively good long‐term prognostic outlook for SCAD patients, many still suffer from significant psychosocial morbidity in the form of recurrent nonischemic chest pain and anxiety or depression. Routine clinical follow‐up of SCAD patients is paramount, and the clinician should be vigilant for signs or symptoms of in‐stent restenosis or recurrent SCAD. However, the evaluation of chest pain can be challenging, and often results in frequent evaluations, diagnostic testing and a substantial psychosocial burden. Further studies are required to explore strategies to improve cardiovascular outcomes.

## 7 CONCLUSIONS

SCAD remains an infrequent cause of ACS in the general population, but accounts for a significant proportion of young women who present with MI, up to 35% in women younger than 50 years. Although both SCAD and atherosclerotic ACS may have a similar presenting symptoms and signs, the disease pathophysiology, predisposing factors, patient demographics and prognostic implications are noticeably distinct. And while we have relatively good evidence behind the rationale for treatment of atherosclerotic ACS, the same is lacking for patients with SCAD. Although large prospective cohort studies have paved the way for improving our understanding of its natural history, randomized trials evaluating the efficacy of pharmacotherapy in SCAD patients are highly anticipated. Furthermore, improving clinician understanding and index of suspicion for this infrequent, but important, cause of morbidity and mortality in young women especially will hopefully improve both immediate and long‐term outcomes in these patients.

## CONFLICT OF INTEREST STATEMENT

Dr. Saw has received unrestricted research grant supports (from the Canadian Institutes of Health Research, Heart & Stroke Foundation of Canada, National Institutes of Health, University of British Columbia Division of Cardiology, AstraZeneca, Abbott Vascular, St Jude Medical, Boston Scientific, and Servier), salary support (Michael Smith Foundation of Health Research), speaker honoraria (AstraZeneca, Abbott Vascular, Boston Scientific, and Sunovion), consultancy and advisory board honoraria (AstraZeneca, St. Jude Medical, Abbott Vascular, Boston Scientific, Baylis, Gore, FEops), and proctorship honoraria (Abbott Vascular, St. Jude Medical and Boston Scientific). The other authors declare no conflicts of interest.

## Data Availability

Data sharing not applicable to this article as no datasets were generated or analyzed during the current study.
